# Neurophysiology of Micturition: a Narrative Review on Preventing Mismanagement

**DOI:** 10.1590/S1677-5538.IBJU.2025.9907

**Published:** 2025-02-22

**Authors:** Ricardo C. Mattos, Luciano A. Favorito

**Affiliations:** 1 Universidade do Estado do Rio de Janeiro Unidade de Pesquisa Urogenital Rio de Janeiro RJ Brasil Unidade de Pesquisa Urogenital, Universidade do Estado do Rio de Janeiro – UERJ, Rio de Janeiro, RJ, Brasil; 2 Hospital Federal da Lagoa Rio de Janeiro RJ Brasil Hospital Federal da Lagoa, Rio de Janeiro, RJ, Brasil

**Keywords:** Urinary Bladder, Neurophysiology, Lower Urinary Tract Symptoms

## Abstract

**Introduction:**

The insidious interrelation between three key factors underscores the critical need to understand the neural control of the lower urinary tract (LUT): the complexity of its functioning, the epidemiology of conditions that can disrupt it, and the nonspecific presentation of related symptoms. This paper examines the importance of understanding neurophysiology of micturition to prevent mismanagement and reduce unnecessary procedures.

**Material and Methods:**

This review focuses on the neurophysiology of the micturition cycle, the epidemiology of major health conditions that affect it, and the nonspecific nature of lower urinary tract symptoms (LUTS) concerning underlying pathologies. The review was conducted in accordance with the guidelines of the Scale for Assessment of Narrative Review Articles (SANRA). Only articles in English were included, while case reports, editorials, and expert opinion pieces were excluded.

**Results:**

The ability of the LUT to store and release urine requires precise coordination and is mediated by a complex network involving the brain, spinal cord, peripheral ganglia, and nerves. Epidemiological data reveal a growing global burden of diseases that impact LUT functioning (LUTF). Moreover, the nonspecific nature of LUTS often leads to diagnostic challenges, and inappropriate treatment strategies.

**Conclusion:**

The interplay between the complexity of LUTF, the widespread prevalence of conditions that can disrupt it, and the nonspecific nature of related symptoms frequently complicate urological decision-making. Overlooking associated neurological factors can result in suboptimal outcomes, diminished quality of life, and serious adverse consequences. A systematic approach is crucial to minimizing the risk of misdiagnosis and mismanagement, especially when considering invasive interventions.

## INTRODUCTION

The insidious interrelation between three key factors underscores the critical need to understand the neural control of the LUT. These factors include the intricate nature of LUTF, the myriad ways in which multiple epidemiologically significant health conditions can disrupt it, and the nonspecific presentation of LUTS, which do not always correspond to the underlying dysfunction. This understanding can serve as a powerful tool for safeguarding patients from iatrogenic harm and protecting physicians from the consequences of inappropriate management.

Comprehending the neurophysiology of micturition is inherently challenging, as patients’ complaints and long-term complications often fail to correlate directly (1). As Turner-Warwick aptly observed, highlighting the complex and sometimes misleading nature of bladder dysfunction, one must not trust the bladder as a witness (2). This complexity emphasizes the importance of proper bladder management in neurologic patients. For instance, in individuals with spinal cord injury (SCI), advances in understanding LUTF have led to significant improvements in care, reducing mortality rates, and diminishing the historical predominance of urinary complications as primary causes of death during early rehabilitation and follow-up (3–5).

The objective of this paper is to evaluate the importance of understanding the neurophysiology of the micturition cycle, with a focus on its role in preventing mismanagement and reducing unnecessary procedures in cases involving neurological diseases.

## MATERIAL AND METHODS

This narrative review focuses on the neurophysiology of the micturition cycle, the epidemiology of key health conditions that affect it, and the specificity of LUTS in relation to underlying pathologies.

The study was conducted in accordance with the SANRA guidelines (6). Relevant literature was retrieved from the PubMed database using Medical Subject Heading (MeSH) terms, with no restrictions on the year of publication.

For the neurophysiology of micturition, we used the search terms "Neurophysiology," "Urination," "Urinary Bladder, Neurogenic," and "Lower Urinary Tract Symptoms."

To gather epidemiological data on "Stroke," "Dementia," "Diabetes Mellitus," "Spinal Cord Injuries," "Intervertebral Disc Disease," "Intervertebral Disc Displacement," "Spinal Stenosis" we used the respective terms combined with "Global Disease Burden," "Epidemiology" and "worldwide" (not a MeSH term).

To assess the specificity of LUTS, the search included terms such as "Urinary Bladder, Underactive," "Urinary Bladder Neck Obstruction," "Urinary Bladder, Overactive," and the terms "Lower Urinary Tract Symptoms," and "overlap" (not a MeSH term).

Only articles written in English were included, while case reports, editorials, and expert opinion pieces were excluded. Only studies deemed significantly relevant to the review's objectives were selected. In addition to the studies retrieved through the systematic search, we also included other literature regarded as fundamental references in this field.

## RESULTS

### NEURAL CONTROL

#### Lower Urinary Tract Functions

The LUT exhibits unique behavior that is highly dependent on central nervous system (CNS), setting it apart from other visceral systems—such as the gastrointestinal and cardiovascular systems—which can maintain basic function even in the absence of extrinsic neural input (7). LUT also has a switch-like or phasic pattern of activity unlike the tonic patterns characteristic of autonomic pathways regulating cardiovascular organs. Furthermore, micturition is also under voluntary control and depends on learned behavior whereas many other visceral functions are regulated involuntarily (8).

The ability of the LUT to store and release urine depends heavily on CNS pathways, requiring precise coordination between the bladder body, bladder neck, urethra, and urinary sphincter. This coordination is mediated by a complex network involving the brain, spinal cord, peripheral ganglia, and nerves. In addition to the nervous system, bladder musculature, urethra, and pelvic floor, structures such as the urothelium, suburothelial and intradetrusorial interstitial cells, and bladder stroma also play significant roles in the micturition cycle (9). These modulators, however, fall outside the scope of this article.

During the micturition cycle, LUT manages urine in two distinct phases: filling and emptying (8). Understanding LUTF is facilitated by correlating its dynamic processes with parameters assessed during urodynamic studies (UDS):

**Filling Phase** - The bladder must accommodate adequate urine volumes without significant increases in pressure, transmit appropriate sensations (neither diminished nor exaggerated), and there must be no involuntary contractions or urinary leakage.

**Emptying Phase** - The bladder must generate contractions of sufficient strength and duration. Simultaneously, the urinary sphincter must relax, without anatomical obstruction or significant post-void residual urine (10).

**Figure 1:**
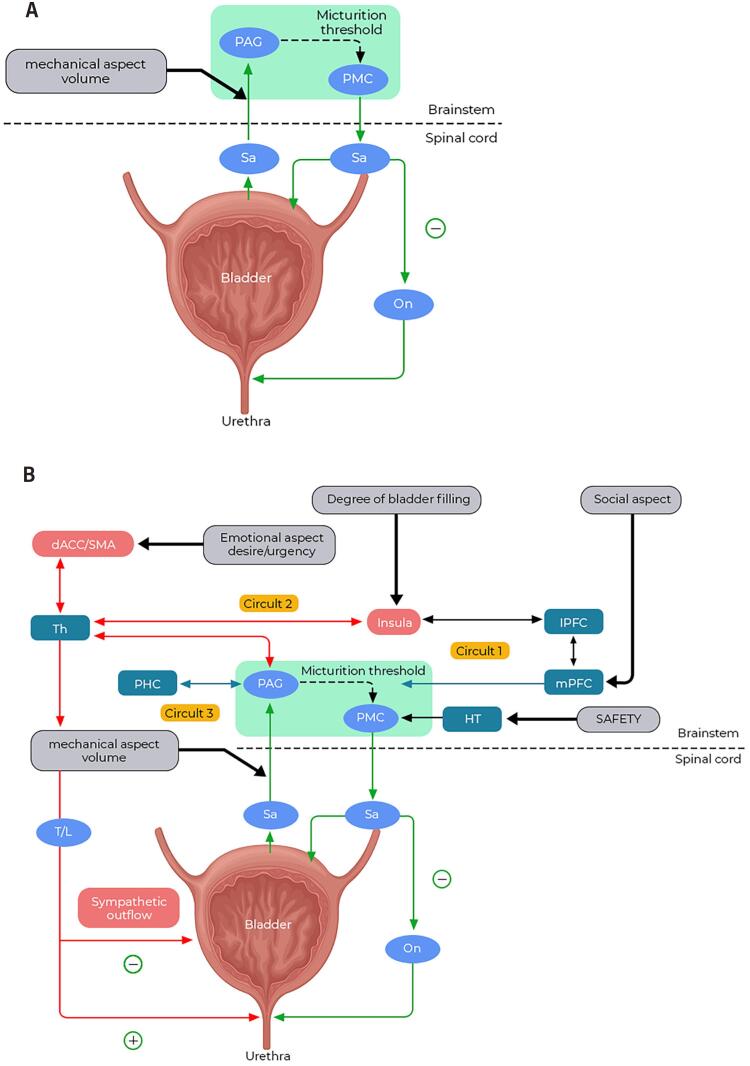
Comparison of the relative simplicity of the voiding reflex pathways and the complexity of higher brain circuit control of the LUT. A) Schematic representation of the voiding reflex, in which the PAG alone can activate the PMC and trigger micturition. B) Higher brain circuits involved in LUT control, with a summary of their respective functions. In circuits one and three, deactivation of the mPFC and parahippocampal regions tends to suppress voiding at the PAG. The hypothalamus, which may be part of circuit three, sends a ‘safe’ or ‘unsafe’ signal to the pontine micturition center. In circuit two, activation of the insula and dACC/SMA generates a strong desire to void or urgency, along with sympathetic motor output to the LUT.

**Abbreviations:** dACC, dorsal anterior cingulate or midcingulate; HT, hypothalamus; lPFC, lateral prefrontal cortex; LUT, lower urinary tract; mPFC, medial prefrontal cortex; On, nucleus of Onuf; PAG, periaqueductal grey; PHC, parahippocampal complex; PMC, pontine micturition center; Sa, sacral parasympathetic region; SMA, supplementary motor area; Th, thalamus; T/L, thoracolumbar sympathetic outflow.

**The Voiding Reflex** - An essential component of LUTF is the spinobulbospinal voiding reflex, also known as the bladder-to-bladder reflex (11) or simply the voiding reflex (Figure-1A). This reflex serves as a central switch between the two micturition cycle phases (12, 13).

During filling, bladder stretch receptors detect increases in bladder volume and transmit sensory signals via the pelvic and hypogastric nerves to the spinal cord. These signals are then relayed to the periaqueductal gray (PAG) in the brainstem.

Once PAG activity reaches a critical threshold—that can be called micturition threshold (7)—it excites the pontine micturition center (PMC), also referred to as the M-region or Barrington's nucleus (14, 15). The PMC is believed to send descending signals to the sacral spinal cord, which, through spinal circuitry, induces urethral sphincter relaxation and, some seconds later (8), bladder contraction (13). This transition, from a completely off mode (storage) to a maximum on mode (voiding), is intrinsic to the very switchlike activity pattern of the pons (16).

In the absence of higher brain control, PAG activation alone can autonomously excite the PMC and trigger the reflex (7, 8, 13). However, under normal circumstances, higher brain regions exert significant control over this process. These regulations will be later described in the Working Model.

#### Filling Phase and Storage Control

Sensory signals from the bladder body are conveyed to the spinal cord via the pelvic and hypogastric nerves (17), while sensory input from the bladder neck and the urethra is transmitted through the pudendal and hypogastric nerves. The afferent components of these nerves include myelinated (Aδ) and unmyelinated (C) fibers (8).

**Afferent Nerve Activity** - The Aδ-fibers respond to passive distension and active contraction (17) providing critical information about bladder filling. Conversely, C-fibers, which are considered ‘silent’ under physiological conditions, are primarily activated by noxious stimuli such as chemical irritation (18) or cooling (19).

**Sympathetic Regulation** - During bladder filling, relaxation of the detrusor muscle is mediated by sympathetic innervation. Norepinephrine activates β3-adrenergic receptors in the bladder wall, promoting relaxation, and α1-adrenergic receptors in the bladder neck and proximal urethra, inducing smooth muscle contraction to enhance closure (20, 21). Sympathetic reflex activity during the filling phase can be triggered by vesical afferent signals in the pelvic nerves and helps the bladder accommodate larger volumes. This can be called the vesicosympathetic reflex, which is inhibited upon reaching the micturition threshold (22).

**External Urethral Sphincter Guarding Reflex** - The pudendal nerve maintains tonic contraction of the external urethral sphincter (EUS), contributing to continence. This tonic activity increases with bladder filling and is mediated, in part, by the guarding reflex, a spinal reflex pathway activated by low-level bladder afferent input, reinforcing EUS contraction (7).

Additionally, contraction of the EUS induces afferent signaling via the pudendal nerve, which, in turn, activates inhibitory interneurons within the spinal cord. These interneurons suppress reflex bladder activity (23) by inhibiting preganglionic neurons and interneurons in the micturition reflex pathway (24). The bladder-to-EUS-to-bladder reflex, along with the vesicosympathetic reflex (7), functions as a negative feedback mechanism to promote urinary continence.

**Pontine Regulation** - During the storage phase, the PMC is typically inactive. However, in another region in the pons, the pontine continence center or pontine urine storage center (PUSC), also called the L-region (8), is active. PUSC, first described neuroanatomically and later by its response to electrical stimulation, not only excites the EUS but also inhibits reflex bladder activity, increasing bladder capacity and the inhibiting the bladder excitatory effect of PMC stimulation (11), probably mediated by stimulation of the urethral sphincter mechanism (25).

**Transition to Voiding** - As bladder volume increases, afferent signals from stretch receptors intensify, informing the brainstem and cortex about bladder fullness. Micturition in the heathy adult occurs only when afferent signals inform enough urine in the bladder, the mechanical requirement, but other criteria must meet, such as safety and social appropriateness (26).

#### The Working Model

Advances in understanding the neural control of micturition, coupled with functional brain imaging studies, have led to the development of a working model based on a framework of circuits that organize brain activity (Figure-1B) (26). While many animal studies have contributed to these advances, a key distinction must be made between micturition in animals and humans: the intention to void. Unlike humans, the intention to void in animals cannot be precisely evaluated, and their micturition models may, in fact, reflect urgency incontinence rather than voluntary control. It is important to note that the working model is a speculative (7) simplification, and its circuits may not function independently.

The working model assumes that three circuits are responsible for the higher control of micturition and ensuring that in a normal adult, micturition occurs only when consciously desired, emotionally safe, and socially appropriate (7).

Circuit one, especially the medial prefrontal cortex (mPFC), assesses social appropriateness. mPFC can either delay or advance voiding by modulating the excitation along the pathway from the mPFC to PAG (26).

Circuit two comprises the anterior insula, the dorsal anterior cingulate cortex (dACC), and the supplementary motor area. The insula (previously assigned to circuit 1) is considered the seat of interoception - the perception of sensations from inside the body (13, 27) and registers the degree of bladder filling, while dACC can create the associated emotion—desire to void or urgency (26). Activation of the supplementary motor area, usually coactivated with dACC, is associated with contraction of the pelvic floor and striated sphincter muscles.

Circuit three encompasses the parahippocampal complex, which may serve as the route through which the PAG monitors bladder behavior and exchanges bladder-related signals with the rest of the brain. This circuit also includes the PAG itself and likely the hypothalamus, which plays a role in ensuring safety for voiding—such as protection from predators—an adaptive mechanism developed through evolution (7, 26).

The PAG activity is modulated by incoming sensory information and communicates with these higher cortical areas. These regions allow conscious decision-making and social appropriateness for voiding. If voiding is deemed acceptable, the PAG activates the PMC.

The PAG plays a pivotal role in voiding, in addition to maintaining homeostasis by regulating systems such as cardiovascular and bowel function, pain, and emotion. Acting as a central hub, the PAG distributes incoming spinal afferent signals to various forebrain regions, generating sensations or motor outputs to prevent incontinence.

In this context, the PAG and the PMC are often considered to function together as the "switch" of the micturition cycle. However, this switch requires modulation, which is thought to be primarily exerted by the forebrain—particularly the medial prefrontal cortex (mPFC). In humans, the mPFC is the site where conscious and voluntary control over voiding is exercised (13), making it the likely seat of executive control (26).

#### Voiding Phase

Once afference signaling has reached micturition threshold in PAG and forebrain modulation favors voiding so that the switch can be done, PMC is then activated, and urine can be eliminated.

Voiding involves parasympathetic activation, trough the pelvic nerve which stimulates detrusor muscle contraction, sympathetic Inhibition, trough the hypogastric nerve that reduces bladder outlet resistance, and somatic relaxation, through the pudendal nerve that reduces external urethral sphincter tone. The synchronized contraction of the detrusor and relaxation of the outlet ensures efficient voiding. Interruptions in this coordination can result in incomplete emptying or incontinence.

Parasympathetic excitation to the bladder is done via acetylcholine (Ach) release, acting on postjunctional muscarinic receptors. In the human bladder, the messenger RNA for all five muscarinic receptor subtypes have been demonstrated (28), with a predominance of M2 and M3 receptors. M3 receptors are believed to be the most important for detrusor contraction (29). When activated, M3 receptors triggers intracellular Ca2+ release; whereas M2 receptors inhibits adenylate cyclase, that contributes to bladder contractions by suppressing adrenergic inhibitory mechanisms which are mediated by β adrenergic receptors and stimulation of adenylate cyclase (30).

Parasympathetic signals can also induce a non-cholinergic detrusor contraction mediated by the released of ATP. ATP excites the detrusor acting on P2X purinergic receptors (30). This route is not important in a healthy adult, but can be involved in some pathological conditions like bladder outlet obstruction, overactive bladder, and interstitial cystitis/bladder pain syndrome (31).

In urethra smooth muscle there is parasympathetic nitric oxide induced relaxation (32) and cessation of adrenergic sympathetic and somatic excitatory inputs to the urethra.

**External Urethral Sphincter** - During the storage phase, increased tonic activity of the EUS is maintained through the spinal guarding reflex and stimulation from the PUSC. However, during micturition, bladder afferent signaling—which contributes to EUS stimulation—is suppressed, resulting in the cessation of the guarding reflex. Additionally, stimulation of the PMC and activation of bulbospinal pathways inhibit EUS motor neurons, leading to sphincter relaxation (33).

#### Modulation on spinal and supraspinal level

In addition to the complex modulatory systems at the autonomic ganglia level, numerous neurotransmitters play key roles in modulating the micturition reflex at both the spinal and supraspinal levels. Many of these neurotransmitters can exert excitatory or inhibitory effects, depending primarily on the receptors they activate. It is important to note that most research on neurotransmitters at these levels has been conducted in animal models.

**Glutamate** - Glutamate is considered the principal excitatory neurotransmitter in the micturition reflex pathway, influencing both excitatory and inhibitory regulation of micturition in the central nervous system (13). Other transmitter systems, such as noradrenergic, dopaminergic, and GABAergic, modulate glutamatergic transmission (34).

On the descending pathway from the PMC, glutamate activates N-methyl-D-aspartate (NMDA) and α-amino-3-hydroxy-5-methyl-4-isoxazolepropionic acid (AMPA) ionotropic receptors, exerting excitatory effects (35). Conversely, activation of metabotropic receptors produces inhibitory effects (36). This inhibitory stimulus on the descending limb of the micturition reflex is also believed to involve the pathway to the EUS (37).

Notably, serotonin and noradrenaline terminals in Onuf's nucleus release glutamate, which induces contraction of the urethral rhabdosphincter. Duloxetine, a norepinephrine and serotonin reuptake inhibitor, enhances urethral rhabdosphincter activity and increases urethral pressures, making it a potential treatment option for stress urinary incontinence (SUI) (38).

At the supraspinal level, glutamate predominantly exerts excitatory effects and is considered essential for voiding function (39).

**Noradrenaline** - The noradrenergic system modulates the micturition reflex by acting as an excitatory input in the efferent limb and an inhibitory input in the afferent limb. The lumbar sympathetic outflow is regulated by α1-excitatory and α2-inhibitory mechanisms (7).

Evidence suggests that α2-adrenoceptor-mediated inhibition and α1-adrenoceptor-mediated tonic facilitation also influence sphincter function, with α2-adrenoceptor-mediated inhibition being the dominant adrenergic modulator of the pudendal nerve reflex (40).

**Serotonin** – Serotonin (5-HT) pathways modulate both the afferent and efferent limbs of the micturition reflex.

Activation of serotonergic neurons on 5-HT receptors in the spinal cord inhibits reflex bladder contractions and sacral efferent pathway firing to the bladder in animal models (41). Descending serotonergic pathways tonically depress the afferent limb of the micturition reflex through 5-HT2 and/or 5-HT3 receptors, enhancing urine storage by facilitating sphincter reflexes in cats and rats (7).

The role of 5-HT1 receptors varies across species, with 5-HT1A receptor agonists increasing bladder capacity in cats (42) but facilitating bladder activity in rats (43).

**GABA and Glycine** - GABA and glycine act as inhibitory neurotransmitters at both spinal and supraspinal levels, increasing bladder capacity, raising the volume threshold for initiating micturition, and reducing voiding pressures in animal studies (7). A GABAergic inhibitory mechanism in the PAG is known to tonically regulate the bladder volume set-point for initiating micturition (44).

**Acetylcholine** - Muscarinic acetylcholine receptors inhibit the micturition reflex in the spinal cord, whereas in the brain, they can exert both inhibitory and facilitatory effects (45).

Nicotinic receptor activation has inhibitory effects on the micturition reflex in the brain but excitatory effects in the spinal cord, enhancing the reflex in rats (46).

**Dopamine** – At the suprapontine level, dopamine plays dual roles: acting on D1-like receptors, it inhibits reflex bladder contractions in cats; acting on D2-like receptors, it facilitates micturition in rats, cats, and monkeys (7).

**Opioids** - Opioid peptides inhibit reflex pathways at both spinal and supraspinal levels, increasing bladder capacity and providing modulatory effects on the micturition reflex (7).

## EPIDEMIOLOGY OF CONDITIONS AFFECTING LUT FUNCTION

The importance of understanding how LUT functions is underscored by epidemiological data showing the growing global impact of diseases that interfere with it. These trends are primarily driven by population aging and growth, but also increases in age-standardized prevalence rates.

Below, we present some of the most common conditions that impact LUTF, categorized as suprapontine, infrapontine-suprasacral, and sacral or more distal conditions.

**Stroke** - According to the Global Burden of Disease (GBD) 2021 study, the age-standardized prevalence of stroke in 2021 was 1,099.31 per 100,000 persons (47). The GBD 2019 study reported an 85% increase in the absolute number of stroke cases from 1990 to 2019, despite a 6% decline in age-standardized prevalence rates during this period (48). This disparity reflects demographic shifts, as population growth and aging contribute to the rising overall burden (48, 49).

**Dementia** - GBD 2019 data revealed significant increases in the global burden of Alzheimer's disease and other dementias between 1990 and 2019, with incidence and prevalence rising by 147.95% and 160.84%, respectively. The age-standardized rates of incidence, prevalence, death, and disability-adjusted life-years have also increased. Women consistently exhibited higher rates, although the rate of increase was more pronounced in men (50).

In high-income countries, the crude prevalence of dementia increased by 119% between 1990 and 2017, despite a 5% decline in global age-standardized incidence rates during the same period. This pattern again suggests that while the risk per individual may be decreasing, the absolute number of cases is rising due to population growth and aging (51). For instance, in Norway, the age-standardized incidence of dementia decreased by 5.4% from 1990 to 2019, while the absolute number of cases rose by 35% (52).

**Spinal cord injury** - The global prevalence of SCI was estimated at 20.6 million individuals in 2019, representing an 85% increase since 1990 (53, 54). However, age-standardized prevalence increased modestly by 5.8% during the same period (53)

The trend is corroborated by other studies, that demonstrate broad regional variations in prevalence rates and a general increase over recent decades (55, 56). These changes are attributed to demographic factors such as population growth and aging, which contribute to a higher number of individuals living with SCI (56, 57).

Despite the rise in absolute numbers, the age-standardized incidence rate of SCI has remained stable, indicating that the prevalence increase reflects demographic changes rather than higher rates of new cases (54, 58). The burden of SCI remains higher in males and older adults, with falls and road injuries being the leading causes (53, 57).

**Figure 2:**
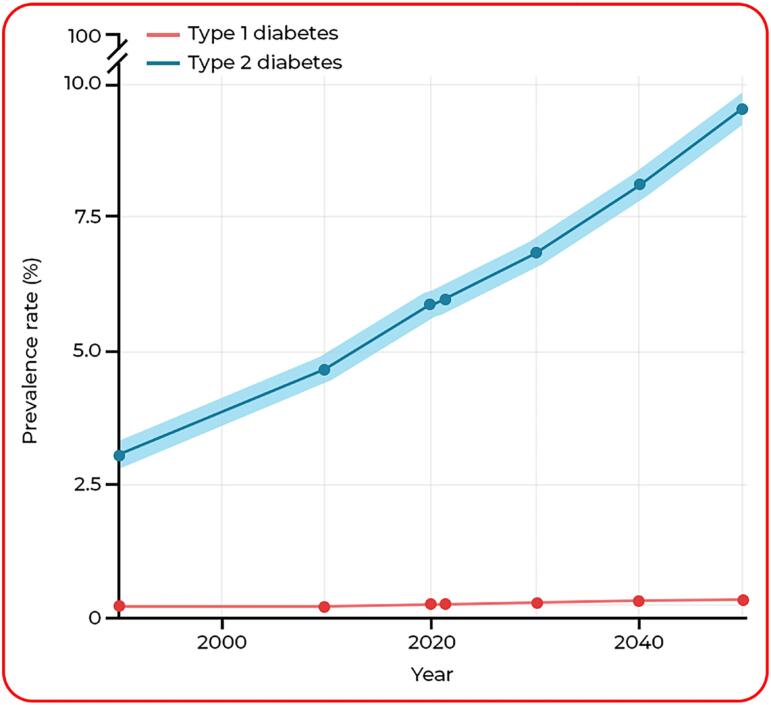
Global age-standardized prevalence of type 1 and type 2 diabetes from 1990 to 2050, including projections. The shaded area represents the 95% uncertainty intervals. The worldwide rise in diabetes prevalence is particularly striking. Between 1990 and 2021, the global age-standardized prevalence of diabetes increased by 90.5%, from 3.2% to 6.1%. Projections indicate a further 59.7% increase between 2021 and 2050, reaching 9.8%, with an estimated 1.31 billion people living with diabetes by 2050. This growth is primarily driven by type 2 diabetes, which is expected to rise by 61.2%, from 5.9% in 2021 to 9.5% in 2050.

**Diabetes** - The worldwide prevalence of diabetes has risen significantly in recent decades. According to the 2021 GBD study, approximately 529 million people were living with diabetes globally in 2021, with an age-standardized prevalence of 6.1%. Projections suggest this number will exceed 1.31 billion by 2050 (Figure-2), with prevalence rates surpassing 10% in regions such as North Africa, the Middle East, and Latin America (59).

Another pooled analysis of 1108 population-representative studies estimated that in 2022, around 828 million adults had diabetes, revealing a substantial increase from 1990 (60). The age-standardized prevalence of diabetes has risen in many countries, particularly in low- and middle-income regions such as South and Southeast Asia, Middle East, and Latin America (60). This trend is largely driven by increases in type 2 diabetes, which is closely associated with rising obesity rates and other lifestyle factors (59, 60).

Diabetes is strongly associated with voiding dysfunction, particularly in patients with polyneuropathy. Early studies reported that 75-100% of patients with documented peripheral neuropathy had underactive bladder (61). A subsequent study using urodynamic evaluations found detrusor underactivity (defined as a Bladder Contractility Index < 100) in 78,8% of diabetic male patients presenting with LUTS (62).

**Lumbar Spine Conditions** - The prevalence of lumbar spine degenerative disease, lumbar disc herniation, and lumbar canal stenosis varies globally and is influenced by factors such as age, gender, and diagnostic criteria.

A Medicare-based study reported overall prevalence of diagnosed spinal degenerative disease of approximately 27.3%, with numbers increasing with age. The study highlights that degenerative findings are common, and the prevalence is likely underestimated due to undiagnosed asymptomatic cases (63).

A study based on magnetic resonance imaging findings reported a prevalence of lumbar disc herniation of 55.1% among individuals with low back pain, with or without lower limb symptoms. The prevalence was higher in those with accompanying lower limb symptoms (82.1%) compared to those with only low back pain (51.6%). The numbers tend to initially increase with age and then decrease in older age groups, with the L4/L5 and L5/S1 segments being most affected (64)

The prevalence of lumbar spinal stenosis in the general population is estimated to be approximately 11% based on clinical diagnoses, with higher rates observed in older adults. It increases with age and affects approximately 103 million people worldwide. Prevalence rates based on radiological diagnoses vary, ranging from 11% in asymptomatic populations to 38% in the general population (65, 66).

Overall, there is a trend of increasing prevalence of these conditions, largely driven by an aging population and improved diagnostic capabilities. However, the estimates should be interpreted with caution due to potential biases in the studies and variations in diagnostic criteria.

## DISCREPANCY BETWEEN SYMPTOMS AND DYSFUNCTION

**Figure 3:**
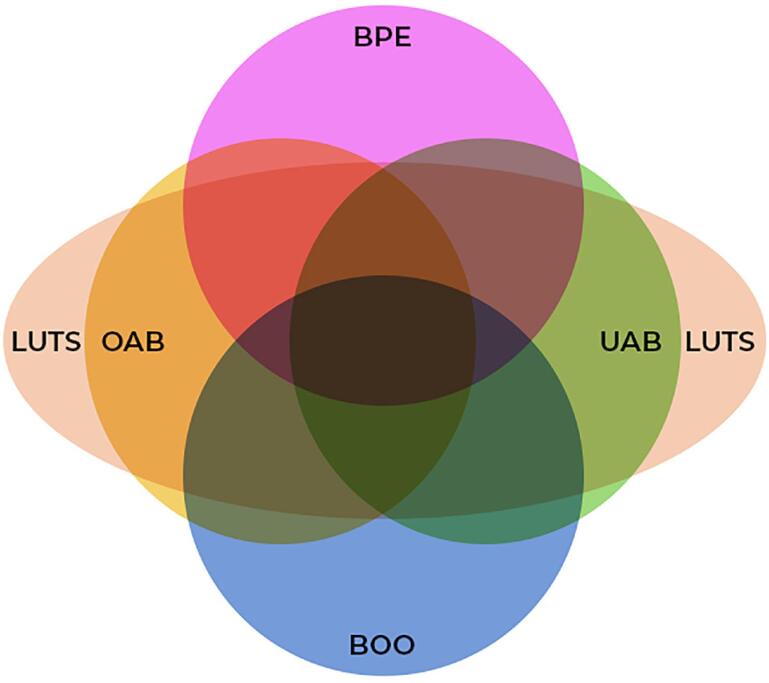
Schematic representation of the overlap between LUTS in the male population. Abbreviations: BPE, benign prostate enlargement; BOO, bladder outlet obstruction; OAB, overactive bladder; UAB, underactive bladder; LUTS, lower urinary tract symptoms.

It is well established that LUTS do not always correlate with the underlying dysfunction, often leading to misdiagnosis and inappropriate management. The discrepancy between symptoms and urodynamic findings has been extensively documented across a variety of conditions affecting LUTF in both male and female patients, spanning different age groups. This dissociation is observed in clinical syndromes such as bladder outlet obstruction (BOO), overactive bladder (OAB), and underactive bladder (UAB) ^(67–76)^. Moreover, these dysfunctions often coexist or are associated with other entities, such as pelvic organ prolapse, further complicating accurate diagnosis (Figure-3).

The diagnostic challenge is particularly pronounced in cases of UAB, where patients typically present with a combination of both storage and voiding symptoms ^(77–79)^.

Nevertheless, the role of LUTF investigation in SUI has been a subject of ongoing debate. A landmark randomized clinical trial (RCT) concluded that "preoperative office evaluation alone is non-inferior to evaluation with UDS in terms of outcomes at one year" and deemed UDS "not justified" in this context (80). Two smaller RCTs reached similar conclusions (81, 82), prompting revisions to influential guidelines (83, 84). Notably, among lower urinary tract conditions, SUI exhibits the highest concordance between clinical complaints and urodynamic findings surpassing conditions such as OAB/urgency urinary incontinence/ detrusor overactivity, voiding dysfunction, and positive post-void residuals (74).

It is essential to recognize, however, that only 22.3% to 39.5% of women presenting with SUI meet the VALUE trial criteria for "uncomplicated" SUI prior to surgery (85, 86). Voiding dysfunction is identified via urodynamic evaluation in approximately 25.6% of patients (86) with higher prevalence in the "complicated" group (32,4%) compared to the "uncomplicated" group (14,7%) (85). Furthermore, urodynamic findings result in the cancellation or modification of planned interventions in nearly 20% of cases (87).

Although not typically classified as neurogenic conditions, loss of bladder capacity and compliance due to radiation or tuberculosis is often overlooked and must be considered in this discussion. These conditions, which, result from direct structural changes in bladder wall, produce symptoms and urodynamic patterns and that overlap with neurogenic bladder dysfunction.

These findings unequivocally demonstrate the complexity of LUT dysfunctions and the potential discrepancies between clinical symptoms and urodynamic findings. Accurate diagnosis is crucial not only to avoid unnecessary surgeries but also to optimize treatment outcomes.

## AVOIDING MISMANAGING

**Figure 4:**
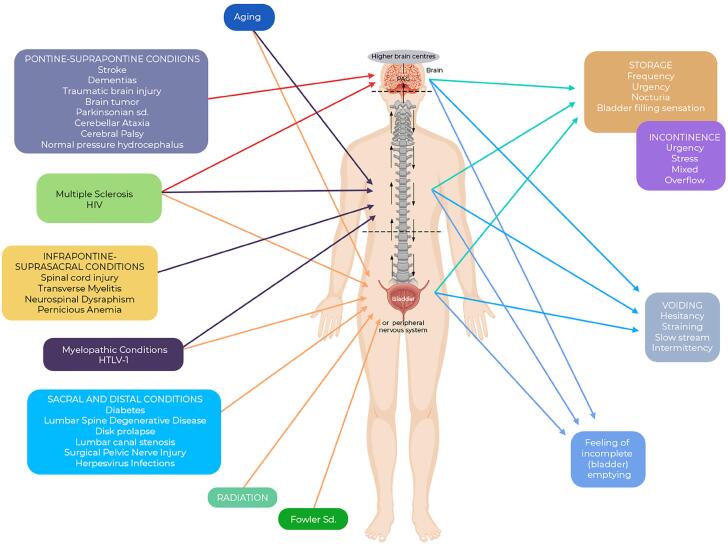
The interplay between three factors: medical conditions affecting LUTF (left), the complexity of neural control of micturition (center), and the non-specificity of LUTS (right), can pose significant diagnostic challenges, increasing the risk of misdiagnosis and inappropriate management. While certain scenarios present with typical clinical patterns (e.g., BOO due to prostatic enlargement often causing voiding symptoms), exceptions exist. For instance, urinary incontinence may result from UAB rather than OAB or stress incontinence, underscoring the need for comprehensive evaluation.

**Abbreviations:** LUTF, lower urinary tract function; LUTS, lower urinary tract symptoms; BOO, bladder outlet obstruction; OAB, overactive bladder; UAB, underactive bladder.

Managing a patient presenting with LUTS who may have a non-urological condition affecting LUTF requires a nuanced understanding of underlying pathophysiological mechanisms, particularly when the condition has not yet been clearly diagnosed (Figure-4).

Patients with undiagnosed or overlooked neurological conditions often do not report key medical histories—such as prior spinal surgery or neurodegenerative diseases—due to a lack of awareness of their potential connection to urinary dysfunction. Furthermore, physicians managing the primary condition may not fully understand its implications for the lower urinary tract. In some cases, the underlying condition affecting LUTF remains undiagnosed, further complicating patient management.

Gender-based diagnostic biases can also impede accurate evaluation. While atypical presentations in women, such as BOO, may result in further investigation more readily, symptoms in males are frequently attributed to prostatic pathology, potentially leading to unnecessary interventions. For instance, performing transurethral resection of the prostate in a patient with a non-contractile bladder is unlikely to improve quality of life. Similarly, treating overflow incontinence caused by a low-sensitivity bladder with a mid-urethral sling in a female patient may exacerbate symptoms rather than resolve them.

Certain conditions, such as hydronephrosis resulting from voiding dysfunction or low-capacity, low-compliance bladders, secondary to radiation or tuberculosis, require careful evaluation. Upper tract dilation may also occur in the case of an underactive bladder, such as following extensive pelvic surgery. Mismanagement in these scenarios can have severe consequences. For example, in a patient with an underactive bladder following extensive pelvic surgery, placing a nephrostomy tube may be detrimental and a simple urethral catheter could suffice.

## CONCLUSION

Caring for patients with neurological conditions affecting LUTF presents significant challenges for urologists. The interplay between three key factors - the complexity of LUTF, the widespread prevalence of conditions that can disrupt it, and the nonspecific nature of related symptoms - frequently complicates clinical decision-making, particularly in complex or atypical cases. Overlooking associated neurological factors can lead to suboptimal outcomes, diminished quality of life, and, in severe cases, serious adverse consequences.

While this discussion has highlighted several prevalent conditions, numerous other disorders impacting LUTF also warrant attention and should not be overlooked. Therefore, it is essential for urologists to consistently consider differential diagnoses beyond more common conditions such as benign prostatic hyperplasia or SUI. Adopting a comprehensive, systematic, and patient-centered approach can significantly reduce the risk of misdiagnosis and mismanagement, especially when invasive interventions are being considered.

## ABREVIATIONS

Ach: = acetylcholine
AMPA: = α-amino-3-hydroxy-5-methyl-4-isoxazolepropionic acid
BOO: = bladder outlet obstruction
dACC: = dorsal anterior cingulate cortex
EUS: = external urethral sphincter
GBD: = Global Burden of Disease
LUT: = lower urinary tract
LUTF: = lower urinary tract functioning
LUTS: = lower urinary tract symptoms
mPFC: = medial prefrontal cortex
NMDA: = N-methyl-D-aspartate
OAB: = overactive bladder
PAG: = periaqueductal gray
PMC: = pontine micturition center
PUSC: = pontine urine storage center
RCT: = randomized clinical trial
RNA: = ribonucleic acid
SANRA: = Scale for Assessment of Narrative Review Articles
SCI: = spinal cord injury
CNS: = central nervous system
SUI: = stress urinary incontinence
UAB: = underactive bladder
UDS: = urodynamic studies
